# Copper concentration in erythrocytes, platelets, plasma, serum and urine: influence of physical training

**DOI:** 10.1186/s12970-021-00426-4

**Published:** 2021-04-07

**Authors:** Víctor Toro-Román, Jesús Siquier-Coll, Ignacio Bartolomé, Francisco J. Grijota, Diego Muñoz, Marcos Maynar-Mariño

**Affiliations:** 1 School of Sport Sciences, University of Extremadura, Avenida de la Universidad s/n, 10003 Cáceres, Spain; 2 Movement, Brain and Health Research Group (MOBhE), Center of Higher Education Alberta Gim´enez (Comillas Pontifical University), Palma de Mallorca, Balearic Islands, Spain; 3 Faculty of Language and Education, School of Sport Sciences, University of Nebrija, Campus La Berzosa, Calle del Hostal, 28248, Hoyo de Manzanares, Madrid, Spain

**Keywords:** Minerals, Trace elements, Training, Platelets, Exercise, Copper

## Abstract

**Background:**

Physical training produces changes in the extracellular and intracellular concentrations of trace minerals elements. To our knowledge, only three compartments have been studied simultaneously. The aim of the present study was to analyze the influence of physical training on extracellular (serum, plasma and urine) and intracellular (erythrocytes and platelets) concentrations of Copper (Cu).

**Methods:**

Forty young men participated in this study. The participants were divided into a training group (TG; *n* = 20; 18.15 ± 0.27 years; 68.59 ± 4.18 kg; 1.76 ± 0.04 m) and a control group (CG; *n* = 20; 19.25 ± 0.39 years; 73.45 ± 9.04 kg; 1.79 ± 0.06 m). The TG was formed by semi-professional soccer players from a youth category with a regular training plan of 10 h/week. All of them had been participating in high level competitions and had trained for at least 5 years. Plasma, serum, urine, erythrocyte and platelet samples of Cu were obtained and analyzed by inductively coupled plasma mass spectrometry (ICP-MS).

**Results:**

The TG showed lower concentrations of Cu in erythrocytes (*p* < 0.05) despite similar intakes. There were no significant differences in Cu concentrations in plasma, serum, urine and platelets although the trend was similar to that observed in erythrocytes.

**Conclusions:**

The assessment of trace element concentrations should be carried out in both extracellular and intracellular compartments to obtain a proper evaluation and to identify possible deficiencies of the element. We believe that additional Cu supplementation is needed in athletes who perform physical training regularly.

## Introduction

Trace mineral elements (TME) are inorganic micronutrients found in several plant and animal foods [1]. TME are known to be involved in a number of biological processes important to health and physical performance [2–4], including oxygen binding and transport, cell energy generation, hormone synthesis, antioxidant activity and muscle contraction [5]. Copper (Cu) is one of the most researched TME [6–8].

Cu is an essential trace mineral in humans. In the organism, it is mainly found in a Cu ^2+^ state although it can also be found in a Cu ^1+^ state [9]. It has similar functions to iron (Fe) and is required for growth, the immune system, energy metabolism, bone mineralization, maturation of red and white blood cells, brain development, transport and metabolism of Fe [2].

This element is also present in a number of cells due to its catalytic role [9]. Besides, it plays an important role as an enzymatic cofactor in cuproenzymes. Cuproenzymes are Cu-dependent oxidases that participate in various reactions that affect numerous physiological processes [10].

Among the different cuproenzymes, cytochrome C oxidase acts as a critical factor in the production of cellular energy. This cuproenzyme is found in the electron transport chain and, after catalyzing the reduction of molecular oxygen to water, is responsible for generating an electrical gradient to create adenosine triphosphate (ATP) [11]. Elsewhere, other cuproenzymes such as ceruloplasmin, cytosolic superoxide dismutase (SOD) and extracellular SOD have an important antioxidant role [12]. SOD transforms the superoxide radical into hydrogen peroxide and oxygen. Cytosolic SOD is found in most cells of the body [9] while extracellular SOD is mainly found in the lungs and plasma [13]. Ceruloplasmin is responsible for transporting Cu and participates in the binding of free Cu ions preventing oxidative damage to muscle tissues [14]. It is also involved in the metabolism of Fe [15]. Other cuproenzymes are linked to reactions for normal brain function, formation of connective tissue in the heart and blood vessels and bone formation [9, 16, 17]. Therefore, as explained above, Cu plays a key role in achieving optimal sports performance.

TME concentrations are usually under strict homeostatic control [18]. However, physical exercise could alter this mechanism and produce changes at extracellular or intracellular levels. In previous research, we observed the influence of physical exercise, both acute and chronic, on Cu concentrations in high-level athletes, amateur athletes and sedentary people in different compartments and modalities [19–23]. In all the studies carried out, we concluded that physical training has a significant influence on the concentrations of TME in the different compartments.

Research studying the influence of physical exercise on Cu concentrations analyses one, two or three compartments [19, 20, 24]. However, to our knowledge, there are no investigation that analyze five compartments simultaneously. We believe that it is important to know the extracellular and intracellular concentrations for studying the body concentrations of TME to assess possible deficiencies in any compartment. Research in the cell compartment is still incomplete, especially in platelets. It is known that the half-life of erythrocytes is approximately 120 days [25], while platelets have a half-life of approximately 10 days [26]. Therefore, we think that while the study of erythrocyte TME informs us of past dietary intake (2 to 3 months earlier), the study of platelets could provide more current information on dietary intake of the mineral (approximately 2 weeks earlier). Therefore, the aim of the present study was to analyze the influence of long-term physical training on extracellular (serum, plasma and urine) and intracellular (erythrocytes and platelets) Cu concentrations.

## Materials and methods

### Participants

Forty young men voluntarily participated in this study. The participants were divided into a training group (TG) and a control group (CG). All the participants had been living in the area of Cáceres, a region in Extremadura (Spain) for at least 24 months before the beginning of the research. The TG consisted of 20 semi-professional soccer players from a youth category belonging to group V of the National Honor Division with a regular training plan of 10 h/week. All of them had been participating in high level competitions and had trained for at least 5 years before the experimental period. The CG consisted of 20 young physically inactive healthy men who did not practice physical activity and had not followed any specific training plan in the previous 6 months. Data on the characteristics of both groups are shown in Table 1.

**Table 1:** Characteristics of the two study groups

	CG (*n* = 20)	TG (*n* = 20)
Age (years)	19.25 ± 0.39	18.15 ± 0.27
Weight (kg)	73.45 ± 9.04	68.59 ± 4.18*
Height (m)	1.79 ± 0.06	1.76 ± 0.04
Muscle (%)	44.22 ± 5.71	49.03 ± 2.56*
Fat (%)	15.64 ± 5.78	9.32 ± 2.76*
VO_2_ max (mL/Kg/min)	45.61 ± 4.95	61.02 ± 4.35**
VE (l/min)	88.34 ± 11.18	120.56 ± 18.79**
Resting heart rate (bpm)	67.31 ± 6.49	54.41 ± 5.29*
Maximum heart rate (bpm)	189.3 ± 7.1	193.8 ± 6.5
Physical activity (MET-hours/weekly)	27.36 ± 4.45	56.13 ± 6.21**

*CG* Control group, *TG* Training group, *VO*_*2max*_ Maximal oxygen consumption, *VE* Expiratory ventilation, *MET* Metabolic equivalent of task; * *p* < 0.05; ** *p* < 0.01

All the participants were informed about the purpose of the study and signed a consent form before enrolling. The protocol was reviewed and approved by the Biomedical Ethics Committee of the University of Extremadura (Cáceres, Spain) following the guidelines of the Helsinki declaration of ethics, updated at the World Medical Assembly in Fortaleza (2013), for research involving human subjects. A code was assigned to each participant for the collection and treatment of the samples in order to maintain their anonymity.

For their inclusion in the study, the participants had to meet the following criteria: be a man, not follow any special diet or take vitamin/mineral supplements, or specific supplementation, medication or over-the-counter medication and not have any injuries or illness during the investigation or at least 6 months before the study. The measurements were carry out at the beginning of the season and were similar to those described by Maynar et al. [20, 23].

### Anthropometric measurements

The morphological characteristics of the participants were evaluated in the morning and under identical conditions. Body height was measured to the nearest 0.1 cm using a wall-mounted stadiometer (Seca 220. Hamburg. Germany). Body weight was measured to the nearest 0.01 kg using calibrated electronic digital scales (Seca 769. Hamburg. Germany) in nude, barefoot conditions. A Holtain© 610ND (Holtain, Crymych, UK) skinfold compass, accurate to ±0.2 mm; a Holtain© 604 (Holtain, Crymych, UK) bone diameter compass, accurate to ±1 mm; and a Seca© 201 (Seca, Hamburg, Germany) brand tape measure, accurate to ±1 mm, were used for the anthropometric assessments. The equations of the Spanish Group of Kinanthropometry [27] were used to calculate the muscle and fat percentage. The anthropometric measurements obtained were height, weight, skinfolds (abdominal, suprailiac, subscapular, tricipital, thigh and leg), bone diameters (bistyloid, humeral biepicondyle and femoral biepicondyle) and muscle perimeters (relaxed arm and leg). All measurements were made by the same operator who was skilled in kinanthropometric techniques, in accordance with the International Society for the Advancement of Kinanthropometry recommendations [28].

### Nutritional evaluation

All participants completed a nutritional survey to guarantee that they were following a similar diet. The survey consisted of a 4-day daily nutritional record, of three preassigned week days, and one weekend day. The participants individually indicated the type, frequency and quantity (in grams) of each food consumed each day, and the nutritional composition of their diets was evaluated using food composition tables [29].

### Physical activity evaluation

Physical activity was assessed using the International Physical Activity Questionnaire – Short Form (IPAQ-SF) Spanish version [30–32]. The questionnaire consists of questions about the frequency and duration of vigorous, moderate and walking activity. A researcher helped the participants to respond. The time spent on vigorous, moderate and walking activity was weighted by the energy spent to produce MET-hours/week. The questionnaire was filled out on the day of the assessments.

### Physical performance test

An exercise test was used to evaluate the performance variables. The trial consisted of an incremental test until exhaustion on a treadmill (Ergofit Trac Alpin 4000, Germany), equipped with a gas analyzer (Geratherm Respiratory GMBH, Ergostik, Ref 40.400, Corp Bad Kissingen) and a Polar pulsometer (Polar® H10, Kempele, Finland). To guarantee a warm-up phase before the test, all participants ran progressively for 15 min, ending at the initial speed of the test. The CG performed 5 min at 6 km/h, 5 min at 7 km/h and 5 min at 8 km/h. The TG ran at 8, 9 and 10 km/h respectively. Then, the participants performed the exercise test. The protocol consisted of running in incremental stages, until voluntary exhaustion (no capacity to keep running) starting at an initial speed of 8 km/h for the CG and 10 km/h for the TG and increasing it by 1 km/h every 2 min, with a stable slope of 1%. All tests were performed in the morning (between 11:00–11:30 a.m.) under similar atmospheric conditions (21–24 °C and 45–55% relative humidity). In the TG, training intensity and volume were reduced on the two previous days applying a regenerative load in order to avoid fatigue in the test.

### Sample collection

At eight o’clock in the morning, 12 mL of venous blood were drawn from each subject in fasting conditions, using a plastic syringe fitted with a stainless-steel needle. Plasma, serum, urine, erythrocyte and platelet samples of Cu were obtained at the beginning of the season. The preparation of the samples was similar to that described by Maynar et al. [20, 23].

For serum, a blood sample of 5 ml was collected in a metal-free polypropylene tube (previously washed with diluted nitric acid) and then centrifuged at 3000 rpm for 15 min at room temperature. Serum was aliquoted into an Eppendorf tube (previously washed with diluted nitric acid) and was left to stand at − 80 °C until further analysis.

For plasma, a blood sample of 5 ml was collected in a metal-free polypropylene tube (previously washed with diluted nitric acid) with ethylenediaminetetraacetic acid (EDTA) and then centrifuged at 1800 rpm for 8 min at room temperature. The platelet rich plasma (PRP) obtained was collected in a metal-free polypropylene tube (previously washed with diluted nitric acid) and centrifuged for 15 min at 3000 rpm and the plasma was aliquoted into an Eppendorf tube (previously washed with diluted nitric acid) and was left to stand at − 80 °C until further analysis. One milliliter of pure water was added to the tube of the concentrate of platelets and stored at − 80 °C.

The erythrocytes were extracted from the rest of the blood and were washed with 0.9% sodium chloride (NaCl) three times. They were then aliquoted into Eppendorf tubes (previously washed with diluted nitric acid) and conserved at − 80 °C until biochemical analysis.

The remaining 2 ml of blood was used for the determination of red blood cells and platelets using an automatic cell counter (Coulter Electronics LTD, Model CPA; Northwell Drive, Luton, UK).

Morning midstream urine samples obtained from all subjects were collected in polyethylene tubes previously washed with diluted nitric acid and frozen at − 80 °C until analysis. Prior to analysis, all samples were thawed and homogenized by shaking.

### Erythrocyte, platelet, plasma, serum and urinary copper determination

The technique used for the determination of Cu was similar to that employed by Maynar et al. [19, 20, 23].

### Sample preparation

Cu analyses were performed by inductively coupled plasma mass spectrometry (ICP-MS). The detection limit was in the range of 1 nmol/L. To prepare the analysis, the organic matrix was decomposed by heating it for 10 h at 90 °C after the addition of 0.8 mL HNO_3_ and 0.4 mL H_2_O_2_ to 2 mL of serum, plasma, urine, erythrocytes and platelets samples. The samples were then dried at 200 °C on a hot plate. Sample reconstitution was carried out by adding 0.5 mL of nitric acid, 10 μL of Indium (In) (10 mg/L) as the internal standard, plus ultrapure water to complete 10 mL.

### Standard and reference material preparation

Reagent blanks, element standards and certified reference materials (Seronorm, lot 0511545, Sero AS Billingstand, Norway) were prepared identically, and used for accuracy testing. Before the analysis, the commercial control materials were diluted according to the manufacturer’s recommendations.

### Sample analysis

Digested solutions were assayed with an ICP-MS Nexion model 300D (PerkinElmer, Inc., Shelton, CT, USA) equipped with a triple quadrupole mass detector and a reaction cell/collision device that allows operation in three modes: without reaction gas (STD); by kinetic energy discrimination (KED) with helium as the collision gas; and in reaction mode (DRC) with ammonia as the reaction gas. Both collision and reaction gases such as plasmatic argon had a purity of 99.999% and were supplied by Praxair (Madrid, Spain). Two mass flow controllers regulated gas flows. The frequency of the generator was free-swinging and worked at 40 Mhz. Three replicates were analyzed per sample. The sample quantifications were performed with indium (In) as the internal standard. The linearity of the calibration curves for In in plasma, serum, urine, platelets and erythrocytes fulfilled the accepted criteria with a value of R^2^ higher than 0.985. The values of the standard materials of this element (10 μg/L) used for quality controls agreed with intra and inter-assay variation coefficients of less than 5%.

### Statistical evaluations

Statistical analyses were carried out with IBM SPSS Statistics 22.0 for Windows (SPSS Inc., Chicago, IL, USA). The normality of the distribution of variables was analyzed using the Shapiro–Wilk test and the homogeneity of the variances with the Levene test. Student’s t-test was used to compare the concentrations between both groups. Effect size (ES) was calculated according to Tomczack and Tomczack [33]. ES of 0.2, 0.4, and 0.8 were considered small, moderate, and large, respectively [34]. A *p* < 0.05 was considered statistically significant. The results are expressed as means ± standard deviations.

## Results

The results of the study are shown below. Table 1 reflects the characteristics of each group. Concerning body composition, there were significant differences in weight, muscle percentage and fat percentage (*p* < 0.05). Besides, there were differences in maximal oxygen consumption (VO_2_ max), expired volume (VE max) and resting heart rate (*p* < 0.05). In addition, physical activity levels were higher in the TG (*p* < 0.01).

Table 2 corresponds to the nutritional intake of each group. No significant differences between groups were observed.

**Table 2:** Nutritional intakes of both groups

	CG (*n* = 20)	TG (*n* = 20)
Energy (kcal/day)	2112.34 ± 345.78	2456.16 ± 504.11
Water (l/day)	1.145 ± 0.241	1.421 ± 0.356
Carbohydrates (g/kg/day)	3.11 ± 1.28	3.98 ± 1.78
Proteins (g/kg/day)	1.25 ± 0.37	1.44 ± 0.41
Lipids (g/kg/day)	1.51 ± 0.47	1.64 ± 0.31
Cu (μg/day)	1931.21 ± 701.33	1832.34 ± 678.43

*CG* Control group, *TG* Training group, *Cu* Copper

The data referring to the total values of erythrocytes and platelets in each group are in Table 3. There were no differences between groups.

**Table 3:** Erythrocyte and platelet values in both groups

	CG (*n* = 20)	TG (*n* = 20)	ES
Erythrocytes (cell 10^12^/L)	4.81 ± 0.72	4.76 ± 0.89	0.14
Platelets (cell 10^9^/L)	190.23 ± 67.13	198.35 ± 60.51	0.17

*CG* Control group, *TG* Training group, *ES* Effect size

Table 4 provides the Cu levels in plasma, serum and urine in both groups. No differences between groups were observed in any extracellular compartment.

**Table 4:** Plasma, serum and urinary concentrations of Cu

	CG (*n* = 20)	TG (*n* = 20)	ES
Plasma (μg/L)	796.69 ± 173.70	765.79 ± 85.45	0.16
Serum (μg/L)	784.89 ± 130.31	778.58 ± 92.17	0.18
Urine (μg/L)	11.50 ± 4.07	10.49 ± 3.12	0.10

*CG* Control group, *TG* Training group, *ES* Effect size

Table 5 shows the absolute and relative concentrations of Cu in erythrocytes and platelets. The TG had lower concentrations of Cu in erythrocytes in absolute and relative values (*p* < 0.05). No significant differences were observed in platelets.

**Table 5:** Relative and absolute values of Cu in erythrocytes and platelets

	CG (*n* = 20)	TG (*n* = 20)	ES
Erythrocytes (μg/L)	468.64 ± 55.49	401.33 ± 51.54**	0.90
Erythrocytes (pg/cell10^−6^)	97.42 ± 18.24	85.39 ± 14.78*	0.57
Platelets (μg/L)	74.60 ± 20.07	65.67 ± 15.33	0.35
Platelets (pg/cell 10^−3^)	0.386 ± 0.114	0.331 ± 0.097	0.28

*CG* Control group, *TG* Training group, *ES* Effect size; * *p* < 0.05; ** *p* < 0.01

## Discussions

The aim of this research was to study the influence of physical training on extracellular and intracellular Cu concentrations. To our knowledge, no studies have been carried out that analyze five compartments simultaneously. Likewise, no investigations analyzing platelet concentrations of TME in athletes have been reported. Most research studying the effect of physical training on TME concentrations refers to one or two specific compartments. This study presents a multi-compartmental approach (plasma, serum, urine, platelets and erythrocytes) to Cu. Although there were no differences in extracellular compartments, significant differences were observed in intracellular compartments. Therefore, given the results, assessments of TME concentrations should not be limited to only the extracellular compartments [18, 23].

The Cu concentrations detected in each compartment are within the ranges reported in other investigations determined with similar techniques [19, 20, 23, 35–37]. As can be seen in Table 1, there were significant differences in body composition, ergo-spirometry and physical activity which reflects the influence of physical training on the TG [38].

It is important to evaluate nutritional intake when assessing the TME in the body. Previous authors reported that assessment of the nutritional status of TME could estimate physical performance [39], highlighting the importance of TME assessment for optimizing physical performance. It is known that food intake is the main source for obtaining Cu [40]. In the present study both groups ingested amounts of Cu in excess of the dietary reference intakes (DRI = 1100 μg/day) [41]. Plasma and red cell values are recognized as indicators of nutritional status for Cu [42]. Our results showed similar estimated intakes of macronutrients, water and Cu (Table 2). Therefore, it could be assumed that the observed differences between groups in Cu concentrations may be due to the influence of physical training. For a better discussion, the analysis of extracellular and intracellular parameters will be examined separately.

No significant differences between groups were observed in the extracellular compartments (plasma, serum and urine) in the current study. In serum, Gropper et al. [43] and Nuviala et al. [44] obtained similar results in women athletes of different disciplines when compared to the CG. Similarly, Dressendorfer and Sockolov [45] did not observe differences between men runners and the CG. Conversely, Maynar et al. [22] showed higher Cu concentrations in anaerobic-aerobic athletes compared to aerobic athletes and the CG despite similar intakes.

Regarding plasma concentrations, Metin et al. [46] reported lower Cu concentrations in soccer players compared to the CG. Equally, Rakhra et al. [47] observed that the highly active group, compared to the moderately active and sedentary group, had lower plasma Cu levels. Conversely, Lukaski et al. [48] did not find significant differences between male swimmers and the CG. However, as in our study, the male swimmers showed lower Cu concentrations. The above study concluded that the nutritional status of Cu is not adversely affected by physical training when dietary intake is adequate. However, they did not analyze the intracellular Cu concentrations. Koury et al. [49] compared Cu concentrations in triathletes, long-distance runners, short-distance runners and short-distance swimmers without observing significant differences among the disciplines. Nonetheless, no CG was present in the previous research. In contrast to the current study, Tuya et al. [19] reported higher concentrations in anaerobic athletes compared to aerobic athletes and the CG. Nevertheless, the mentioned study did not quantify the intake of Cu which could affect plasma concentration.

In urine, several studies found a higher Cu excretion in athletes when compared to the CG at baseline [20, 21, 44, 50]. Muñoz et al. [21] reported that the rise in urinary Cu observed in the previous studies could be related to the biological metabolism of this mineral induced by physical training. Perhaps, the reduced Cu excretion is due to a low Cu flow which would be related to what was observed in plasma and serum.

The low Cu concentrations in plasma and serum observed in this study could be due to different consequences of regular physical training. One consequence could be an increased sweat loss [48, 51], or elevated activity of the antioxidant defense mechanisms. The higher antioxidant activity would increase the use of Cu for the synthesis of Cu-Zn (Zinc) SOD and ceruloplasmin [47]. In this respect, Koury et al. [49] observed inverse relationships between serum Cu and Cu-Zn SOD concentrations (*r* = − 0.32; *p* = 0.02). In addition, previous researches have reported lower concentrations of ceruloplasmin in athletes compared to CG [43, 52]. Besides, lower Cu concentrations in the TG could be related to rises in cytochrome C oxidase activity which are increased after endurance training [53, 54]. This would be in parallel with the aerobic component of soccer and the training volume rise at the beginning of the season [55]. Additionally, the possible difference in concentration and mitochondrial function between groups (higher in the TG), caused by physical training, would increase the use of Cu to maintain cytochrome C oxidase activity [56–58].

Concerning intracellular concentrations, lower concentrations of Cu in erythrocytes were observed in the TG (*p* < 0.05). This would indicate that in the previous 2–3 months there may have been a low intake of Cu in the diet. There were no significant differences in platelets, which could indicate that in the previous 2 weeks Cu intake was not deficient in any group. However, the platelet Cu concentration in the TG followed a similar trend to that of erythrocytes, which are in parallel with those observed by Maynar et al. [23]. Moderately trained and highly trained athletes showed lower concentrations compared to the CG. Furthermore, they observed an inverse relationship between the degree of training and the concentrations in erythrocytes of Cu (*r* = − 0.790; *p* = 0.000). Similarly, Singh et al. [59] reported lower concentrations of Cu in red blood cells in women athletes. No studies have been found in athletes regarding platelets. Nevertheless, they were within the range reported by Catalani et al. [36].

Reduced Cu concentrations in erythrocytes would be related to the role of cuproenzymes in antioxidant activities, such as in the extracellular compartments. Erythrocytes are known to contain Cu-Zn SOD [23]. In this sense, previous studies reported that the enzymatic activity of Cu-Zn SOD is higher in athletes than in sedentary subjects [60, 61]. However, while the level of training increases, Cu-Zn-SOD activity decreases [60]. Other authors concluded that the decrease in Cu in erythrocytes does not appear to be related to a modification in Cu intake, but rather reflects a redistribution in tissues [62]. Another possible explanation for the low erythrocyte Cu values could be related to a deficient absorption of Cu due to an interaction with other nutrients such as Zn [63–65]. Regarding Cu concentrations in platelets, to our knowledge, no studies in athletes have been reported. Other authors have analyzed the concentrations of TME in platelets with the neutron technique [66]. The studies found that compare platelet concentrations of TME were conducted on people with pathologies [67, 68]. Further research on platelets in athletes is needed to confirm and compare results.

Some limitations of the study should be noted: first, the small single-sex sample, thus, additional participants are needed to confirm results; second, a cross-sectional study was conducted by obtaining samples at one moment during the sports season in the TG; third, complementary Cu data such as ceruloplasmin or SOD were not analyzed. Finally, future research should explore gender differences as well as conduct longitudinal studies at different moments in the sports season. Moreover, future researchers could complement the present results with data on metabolic and antioxidant status.

## Conclusions

Our study reveals lower erythrocyte concentrations of Cu in people performing physical training on a regular basis, despite similar intakes. Nevertheless, there were no significant differences in extracellular (plasma, serum and urine) or platelet concentrations, although the trend was similar to that observed in erythrocytes. The results in erythrocytes could be due to nutritional deficiencies of Cu in previous months.

As a consequence of the results obtained, we suggest that the condition of TME should be analyzed in both extracellular and intracellular compartments in athletes who perform regular physical training in order to obtain a complete assessment. We suggest regular assessments of Cu concentrations to avoid deficiencies in athletes. Moreover, we encourage researchers to assess TME concentrations in platelets as this could provide information on the current intracellular concentrations with respect to erythrocytes that give us information from previous weeks or months.

## Abbreviations

ATP: Adenosine triphosphate
Cu: Copper
CG: Control group
EDTA: Ethylenediaminetetraacetic acid
Fe: Iron
ICP-MS: Inductively coupled plasma mass spectrometry
IPAQ: International physical activity questionnaire
MET: Metabolic equivalent of task
PRP: Platelet rich plasma
RDI: Recommended daily intake
Rpm: Revolutions per minutes
SOD: Superoxide dismutase
TG: Training group
TME: Trace mineral elements
VE: Expired volume
VO_2max_: Maximal oxygen consumption
Zn: Zinc
